# Computer Simulations of Lipid Nanoparticles

**DOI:** 10.3390/nano7120461

**Published:** 2017-12-20

**Authors:** Xavier F. Fernandez-Luengo, Juan Camacho, Jordi Faraudo

**Affiliations:** 1 Institut de Ciència de Materials de Barcelona (ICMAB-CSIC), Carrer dels Til.lers s/n, Bellaterra, E-08193 Barcelona, Spain; xavierfdezlflores@gmail.com; 2 Departament de Física, Facultat de Ciències Universitat Autònoma de Barcelona, Campus de la UAB, Bellaterra, E-08193 Barcelona, Spain; Juan.Camacho@uab.cat

**Keywords:** lipid nanoparticles, molecular dynamics, Martini force field, self-assembly, softmatter

## Abstract

Lipid nanoparticles (LNP) are promising soft matter nanomaterials for drug delivery applications. In spite of their interest, little is known about the supramolecular organization of the components of these self-assembled nanoparticles. Here, we present a molecular dynamics simulation study, employing the Martini coarse-grain forcefield, of self-assembled LNPs made by tripalmitin lipid in water. We also study the adsorption of Tween 20 surfactant as a protective layer on top of the LNP. We show that, at 310 K (the temperature of interest in biological applications), the structure of the lipid nanoparticles is similar to that of a liquid droplet, in which the lipids show no nanostructuration and have high mobility. We show that, for large enough nanoparticles, the hydrophilic headgroups develop an interior surface in the NP core that stores liquid water. The surfactant is shown to organize in an inhomogeneous way at the LNP surface, with patches with high surfactant concentrations and surface patches not covered by surfactant.

## 1. Introduction

The continuous increase in computer power and the development of new methods for large-scale Molecular Dynamics Simulations (MD) have made possible its use as a kind of computational microscope to study nanoscale systems and processes [1]. Molecular modelling is particularly useful in new fields, in which new basic concepts, qualitative or quantitative predictions or even suggestions are needed. One such field in which molecular modelling is particularly useful is the development of new nanomaterials for drug delivery applications. The objective of these nanomaterials is to solubilize non-soluble active pharmaceutical ingredients and, in more advanced formulations, to provide a vehicle that maximizes efficiency by delivering the active ingredient to a specific target (tissue or cell) [2]. In this case, molecular modelling can provide useful insights that help rational design of the new materials. For example, in a recent combined experimental and simulation work [3], we designed a new, highly efficient nanomedicine (based on liposomes) for a rare disease. Using large-scale molecular dynamics simulations, we identified the reason for its high efficiency as linked to the way in which the active component (an enzyme) is positioned at the surface of the nanocarrier. Now, there are many computational studies of drug delivery nanomaterials such as liposomes, micelles, dendrimers and many others that provide valuable insights on these materials. However, other important drug delivery nanomaterials remain unexplored from the point of view of molecular simulations. This is the case for the so-called lipid nanoparticles [4], which we propose to explore in the present paper using molecular dynamics simulations of a coarse-grain molecular model.

Lipid nanoparticles (LNPs) represent an alternative drug delivery nanomaterial, which is made of a single lipid (such as tripalmitin) or a mixture of lipids and stabilizing surfactants [4,5]. Depending on the composition, stabilities of three years or more have been reported [6], although many formulations tend to particle aggregation and gel formation, depending on the composition and storage temperature. Typically, LNPs are stored at temperatures low enough to ensure a solid phase for the lipids (typically 5 ${}^{\circ }$C–10 ${}^{\circ }$C) [6,7,8], which guarantees a negligible diffusivity of lipophilic active principles trapped inside the LNPs. At higher temperatures (room temperature and human body temperature), the lipids inside the LNPs are believed to be in liquid phase. For example, in the case of LNPs made of tripalmitin lipid and Tween 20 surfactant [8], calorimetric studies show a melting temperature for the lipid inside the LNP about 25 ${}^{\circ }$C, whereas, in pure (bulk) lipid, the melting temperature is 47 ${}^{\circ }$C at 1 atm of pressure.

There is a wealth of thermodynamic and stability data on LNPs [6,7,8] , but little is known about the molecular organization of their components inside the LNPs. In fact, there are very few molecular modelling studies on these systems [9] and their design (decision on lipid and surfactant composition for example) is essentially made by trial and error. Our objective here is to employ molecular dynamics simulations to investigate how lipid molecules organize themselves inside these LNPs and how surfactants cover these LNPs. To this end, we will employ a coarse-grain (CG) methodology that will allow us to perform simulations of full LNPs, although of small size (5–10 nm). CG models focus on essential features, averaging over less important atomistic details. In that way, these models provide substantial advantages with respect to more detailed, fully atomistic models, both from computational (faster calculations, possibility of reaching longer time scales and larger length scales) and conceptual (easier to interpret) perspectives [10,11]. Combined with advances in computational power, CG models allow researchers the possibility of performing molecular simulations in problems that were previously inaccessible. There are many CG models suitable for simulation of soft matter systems and organic molecules (see [10] for a review). Here, we have decided to employ the popular Martini model, which is especially suitable for the simulation of self-assembly of organic molecules [11,12,13].

**Figure 1:**
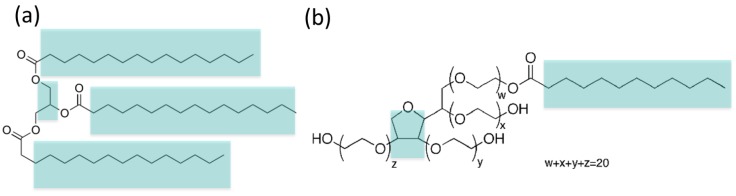
Chemical structures of the amphiphilic molecules considered in this work: (**a**) tripalmitin lipid and (**b**) Tween 20 surfactant. We indicate their hydrophobic regions in cyan colour (the remaining regions of the molecules are hydrophilic).

We will consider in particular LNPs made of tripalmitin lipid covered with Tween 20 surfactant (see Figure 1), for which there are available detailed experimental studies [8]. These molecules are also interesting from the conceptual point of view, as they have in their chemical structure a combination of hydrophobic and hydrophilic regions as indicated in Figure 1. They are more complex than simple phospholipids or classic surfactants (such as micelle forming SDS or CTAB surfactants for example), which only have a single hydrophilic headgroup and one or two hydrophobic tails. It is thus interesting, from a purely theoretical point of view, to study their self-assembly behaviour. As we will see, our results reveal interesting structural features (presence of water inside the LNPs, inhomogeneity of surfactant distribution), which have an impact in our understanding of these nanomaterials.

The paper is organized as follows. In Section 2, we develop a CG model for tripalmintin and Tween 20 compatible with the Martini force field, and we describe the molecular dynamics simulations performed in this work. In Section 3, we present our results and discussion. We close the paper with our Conclusions.

## 2. Computational Methods

### 2.1. Coarse-Grain (MARTINI) Model of Tripalmitin and Tween 20

The Martini model is based on a building block principle, in which groups of atoms are replaced by a single bead that interacts with other beads with an effective interaction designed to maintain the chemical specificity. Typically, the model maps 4–5 heavy atoms (C,N,O) and their associated hydrogen atoms to a single interaction centre, although some specific cases (e.g., molecules with rings) require a treatment with higher resolution.

In the Martini model, there are four generic types of beads (Q = charged, P = polar, N = nonpolar and C = apolar) and, within each main type, subtypes are distinguished by a letter denoting the hydrogen bonding capabilities (d = donor, a = acceptor, da = both, 0 = none) or by numbers indicating the degree of polarity (1–5). In the Martini model, each bead has a mass of 72 amu, irrespective of its type. Precise definitions of the different beads of the Martini model and their size and interactions can be found in Refs. [11,14] (see Ref. [14] for the full interaction matrix between all bead types).

Following the prescriptions of the Martini model, we have developed a CG model for the tripalmitin lipid and from the Tween 20 surfactant starting from their atomistic structure.

**Figure 2:**
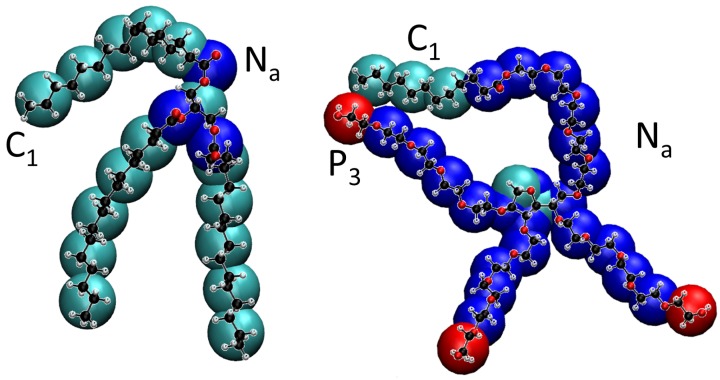
Modelling of tripalmitin lipid (**left**) and Tween 20 surfactant (**right**). We show the correspondence between the beads of the CG Martini representation with the all-atomic model (in CPK representation). In the all atomic model, hydrogen atoms are shown in white, carbons in black and oxygen atoms in red. In the Martini coarse-grain model, the C${}_{1}$ beads are shown in cyan, N${}_{a}$ beads in blue and P${}_{3}$ beads in red.

Tripalmitin is a triglyceride derived from a fatty acid (palmitic or hexadecanoic acid). It is an amphiphilic molecule; its ester groups give the molecule its hydrogen bonding capability while the alkylic tails have hydrophobic character (see Figure 1). According to the definition of Martini beads [14], we have mapped the alkylic tails and the headgroup linker to 16 hydrophobic beads of type C${}_{1}$ (the most apolar bead type allowed in Martini) and each of the three ester groups to one N${}_{a}$ bead, as shown in Figure 2 left. After this coarse-grain procedure, we have replaced the atomic model with 155 atoms by a CG model with only 19 beads.

Tween 20 (also known as polysorbate 20, see Figure 1) is a polysorbate-type nonionic commercial surfactant widely employed as a detergent and emulsifier in many domestic, scientific and pharmaceutical applications. As in the previous case, we started from an all-atomic model, and, following the Martini protocol, we assigned CG beads with standard Martini types (see Figure 2 right). In this case, the molecule has a central ring which requires a careful modelling, as described in [11]. Since the geometry of the ring has to be maintained, in this case the mapping was made assigning 1 heavy atom plus their hydrogen atoms to a single bead (which was of N${}_{a}$ or C${}_{1}$ type, see Figure 2). The mappings for the rest of the molecule simply follow the standard Martini rules, so we replaced groups of atoms by beads of types P${}_{3}$ (polar with a level 3 of polarity), N${}_{a}$ and C${}_{1}$ as shown in Figure 2. In that way, the atomistic model containing 198 atoms was transformed into a CG model with 30 CG beads. Once the model for tripalmitin and Tween 20 was developed, we have built structure and coordinate files for the beads from the atomistic coordinates using the coarse-graining tools implemented in the Visual Molecular Dynamics (VMD) program version 1.9 [15]. These files (in pdb, top and psf formats) are available from the authors under request.

### 2.2. Methodology for Molecular Dynamics Simulations

After developing the models for the molecules to be simulated, we can perform simulations of systems containing large numbers of molecules. Here, we will consider simulations of the self-assembly of tripalmitin molecules in water, giving rise to NPs of different sizes. After that, we have considered the effect of adding Tween 20 surfactant. In all of our simulations, we have employed the Molecular Dynamics (MD) technique. Molecular Dynamics (MD) simulations are based on the numerical solution of the Newtonian equations of motion of a molecular system constrained to the given thermodynamic conditions. In our case, we will consider always 310 K and 1 atm, which are the conditions of interest for biological applications of LNP.

All MD simulations reported here were performed using the free NAMD software [16], version 2.9. The equations of motion were solved with a Δt=20 fs time step, which is a conservative selection to ensure stability of ring molecules (more aggressive time steps of 40 fs are usually employed in Martini MD simulations (see [11])).

All molecules were described using the Martini force field. For tripalmitin and Tween 20 molecules, we have employed the models described earlier in this section. For water molecules, we have employed the standard Martini CG water model [11] that replaces water molecules (which have strongly anisotropic hydrogen bonding interactions) with a mixture of polar P${}_{4}$ spherical beads and special anti-freezing (AF) beads. One P${}_{4}$ bead corresponds to four water molecules and the number of AF beads is one for each ten P${}_{4}$ beads. The highly polar, highly interacting P${}_{4}$ beads when left alone tend to form isotropic crystals at room temperature, which are disrupted by the AF beads. With this mixture of two beads, one obtains a highly interacting liquid at room temperature that resembles water. More details on the Martini water model can be found in Refs. [11,14].

All interactions (intramolecular and intermolecular) were computed employing the Martini force field equations for bonds, angles, dihedrals and Lennard–Jones interactions as implemented in NAMD. Lennard–Jones interactions were truncated at 1.4 nm employing a switching function starting at 1.0 nm as usual in NAMD [16]. Periodic boundary conditions in all directions were employed in all our simulations. In all simulations, the temperature was kept constant at 310 K using the Langevin thermostat and the pressure adjusted to 1 atm employing the Nosé–Hoover–Langevin piston [16].

The preparation of the MD simulations and the analysis of the MD trajectories have been made using the VMD program [15] and home-made tk/tcl scripts running in VMD. All snapshots reported in this paper have been made using VMD. The self-assembly of the molecules during the simulations has been characterized by calculating the radius of gyration ($R_{g}$), defined as:

(1) $$R_{g}^{2}\left(t\right)=\frac{\sum_{i=1}^{N}m_{i}{\left({\vec{r}}_{i}\left(t\right)-{\vec{r}}_{CM}\left(t\right)\right)}^{2}}{\sum_{i=1}^{N}m_{i}},$$

where ${\vec{r}}_{CM}$ is the centre of mass of the *N* molecules. On average, the LNP to be studied are expected to be spherical, but it could be possible that typical LNP realizations may deviate from a perfect spherical shape. In order to characterize a possible deviation from a spherical shape, we have also calculated the three principal radii of gyration $R_{1}$, $R_{2}$ and $R_{3}$, which should be equal for a sphere (in general, we have $R_{g}^{2}=R_{1}^{2}+R_{2}^{2}+R_{3}^{3}$). Instead of reporting the three principal radii of gyration, we report the asphericity parameter Δ defined as:

(2) $$\Delta =\frac{3}{2}\frac{\sum_{i=1}^{N}{\left(R_{i}-R_{m}\right)}^{2}}{{\left(R_{1}+R_{2}+R_{3}\right)}^{2}},$$

where $R_{m}=\left(R_{1}+R_{2}+R_{3}\right)/3$ is the average radius. The parameter Δ is always between 0 and 1, with Δ=0 corresponding to a perfect sphere. This definition for Δ has been employed previously to characterize deviation from spherical shape in polymers, proteins and DNA chains [17].

### 2.3. Description of Simulated Systems

We have performed MD simulations of five different systems, with different compositions in order to obtain LNPs of different sizes and to study the effect of the addition of surfactant. The composition of all simulated systems is given in Table 1.

**Figure 3:**
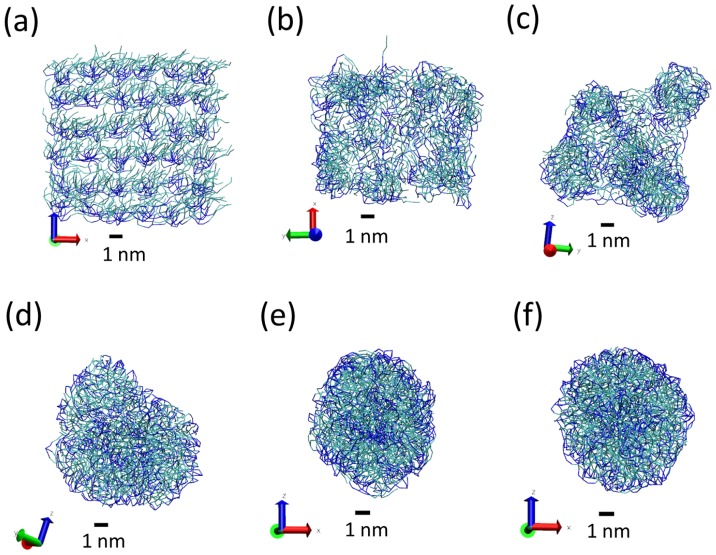
Snapshots of simulation S2 of a tripalmitin LNP at different simulation times. The initial configuration for MD simulations (*t* = 0), generated after energy minimization, is shown in (**a**) subsequent evolution from MD simulations is shown in the other panels: (**b**) 30,000 time steps (*t* = 0.6 ns), (**c**) 90,000 time steps (*t* = 1.8 ns); (**d**) 1.9 ×$10^{5}$ time steps (*t* = 3.8 ns); (**e**) 2.9 ×$10^{5}$ time steps (*t* = 5.8 ns); (**f**) 3.9 ×$10^{5}$ time steps (*t* = 7.8 ns). Tripalmitin molecules are displayed in bond representation, with hydrophobic C${}_{1}$ beads in cyan and hydrophilic N${}_{a}$ beads in blue. Water is not shown for clarity. A scale corresponding to 1 nm is shown to help the reader. The size of the simulation box (containing the solvent and the lipids) is much larger than the structures shown here and it is not indicated in the snapshots.

The simulations of the different systems were prepared as follows. First, we generate the coordinates for the lipid and also surfactant molecules (if any) in a periodic array. Then, we solvate the system by adding water molecules (both P${}_{4}$ and AF beads) using the VMD program. In this way, we have an array of molecules inside a big solvation box, always much larger than the dimensions of the initial array of molecules. After solvation, an energy minimization with NAMD is performed before running the actual MD simulations. During the MD simulations, we monitor physical quantities (such as the size of the simulation box, energy, …) to ensure that the simulated systems reach the equilibrium state. In all three cases, we observe that tripalmitin molecules self-assemble spontaneously into a single, well defined LNP, which also includes the surfactant molecules (if included in the simulation). The self-assembly process is illustrated in Figure 3 for simulation S2. After a few ns of simulation, the lipid molecules are assembled into a non-spherical structure, which becomes more compact with time and finally becomes spherical. For example, this process takes about 4 ns for simulation S2 (which has 216 lipid molecules, see Figure 1). In all cases, we performed the MD simulations for times much larger than the times required for the formation of the LNP. The total simulation times are indicated in Table 1. All results reported in this paper correspond to averages over configurations corresponding to the equilibrium state, after formation of the LNP.

It is also worth emphasizing that, in this work, all reported times are nominal times, computed as usual as the number of steps performed in the simulation times the time corresponding to a time step Δt. However, one has to keep in mind that, in general, when using CG models (and when using the Martini model in particular), the time scales of the model require a careful interpretation since they do not exactly correspond to actual physical time scales. This is due to various effects such as a faster diffusion of water and other molecules as compared with atomistic models and real molecules. In the case of Martini, it is believed that a good estimation of the actual physical time (i.e., the one related to experiments) can be obtained by multiplying nominal time by a factor of 4 [11]. However, in the absence of more conclusive studies on this matter, we prefer to indicate only nominal times always in this paper.

## 3. Results and Discussion

### 3.1. Tripalmitin Nanoparticles of Different Sizes

We have performed simulations of tripalmitin molecules in water (at T = 310 K and 1 atm of pressure) considering three different amounts of tripalmitin molecules in order to obtain LNPs of different sizes (simulations S1, S2 and S3 in Table 1). As explained in Section 2, in all cases, we observe that tripalmitin molecules self-assemble spontaneously into a single, well defined LNP. In order to characterize the size and shape of the obtained LNPs, we have computed the radius of gyration $R_{g}$ and the asphericity parameter Δ as defined in Equations (1) and (2) for all simulations. The results are given in Table 2.

The radius of gyration $R_{g}$ is 2.5 nm for the smallest particle and 4.6 nm for the largest one. In addition, the results are consistent with a particle volume proportional to the number *N* of lipids per nanoparticle; using the values of $R_{g}$ to estimate the volume of the LNPs, we obtain a number density of ≈1 lipid/nm${}^{3}$ in the three cases (see column 4 in Table 2). The values obtained for the asphericity parameter are of the order $\Delta \approx 10^{-3}$–$10^{-4}$ indicating that the shape of the LNPs is spherical (Δ=0 corresponds to a perfect sphere and Δ=1 corresponds to a rod).

**Figure 4:**
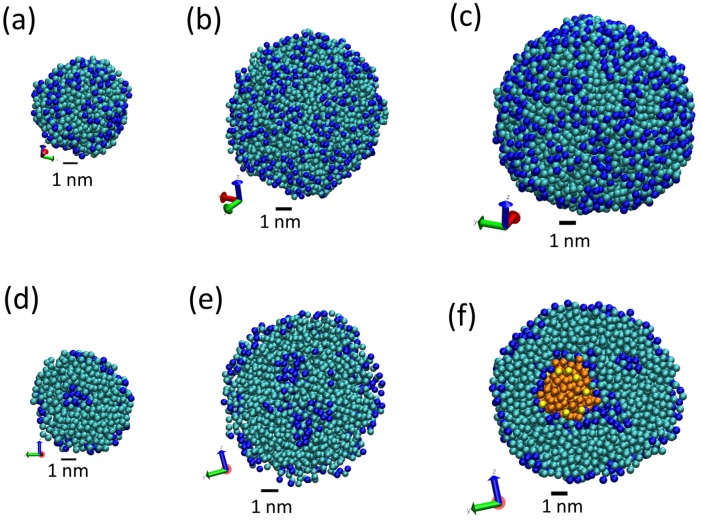
Snapshots from simulations S1 (**a**,**d**), S2 (**b**,**e**) and S3 (**c**,**f**) illustrating the different structures of LNPs of different sizes. Snapshots (**a**–**c**) show the full LNP and snapshots. Snapshots (**d**–**f**) show a cross section of the LNP. All Martini lipid model beads are shown as spheres (hydrophilic N${}_{a}$ beads are shown in blue and hydrophobic C${}_{1}$ beads are shown in cyan). We also show water molecules present inside the LNP (P${}_{4}$ beads in orange and AF beads in yellow). Water molecules outside the LNPs are not shown.

**Figure 5:**
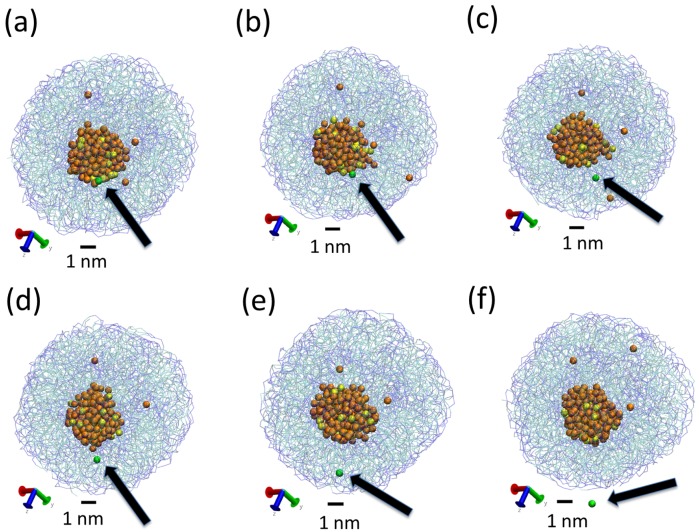
Snapshots from simulation S3, showing the exchange of water from inside the LNP to the solvent. Tripalmitin lipid molecules are displayed in bond representation using transparent colours to facilitate visualization of inner water. Water molecules present inside the LNP are shown as spheres (P${}_{4}$ beads in orange and AF beads in yellow). Water molecules outside the LNPs are not shown. A particular water bead (of P${}_{4}$ type) is shown in green (see also the arrow) to follow its movement during the simulation. Panels from (**a**) to (**f**) correspond to snapshots taken at intervals of 10 ns so the total time from (**a**) to (**f**) is 50 ns. In panel (**a**), the water bead is at the surface of the inner water droplet. In (**b**), the bead detaches. In panels (**c**,**d**), the water bead diffuses inside the lipid core of the LNP and finally it exits from the LNP at (**f**).

The obtained LNPs are shown in Figure 4. In this figure, we can see interesting features of the organization of the lipids in the LNPs. In panels (a), (b) and (c), we can see the organization found at the surface of the simulated LNPs. We see that at the LNP surface we have both hydrophobic and hydrophilic groups. This is in marked contrast with classical self-assembly amphiphilic colloids such as micelles, vesicles or liposomes, in which only the hydrophilic head groups are exposed to the water solvent. In these LNPs, there is a lack of enough hydrophilic groups to completely cover the surface of the particles. We think that this effect is due to the particular structure of the tripalmitin molecule. As seen in Figure 1, this molecule has a large, bulky hydrophobic region and small hydrophilic groups separated by a hydrophobic region. Therefore, tripalmitin does not have a well-defined hydrophilic headgroup as other amphiphilic molecules have (for example, phospholipids). When a tripalmitin molecule exposes hydrophilic regions to water, it also exposes hydrophobic regions in contact with water. In addition, the cross sections shown in panels (d), (e) and (f) of Figure 4 indicate the presence of both hydrophobic and hydrophilic groups inside the LNP. Tripalmitin molecules inside the LNP can arrange their small hydrophilic groups to be in mutual contact inside the LNP and avoid the penalty of exposing hydrophobic regions in contact with water. In simulations S1 and S2 (panels (d) and (e)), the hydrophilic groups inside the LNP are organized in small clusters. In the case of the larger LNP (simulation S3, panel (f)), most of the hydrophilic groups located inside the LNP surround a water droplet located inside the LNP core. The diameter of the water droplet located inside the LNP is about 2–3 nm, as seen in Figure 4f. The average number of water beads inside the LNO in simulation S3 is 170 P${}_{4}$ beads and 18 AF beads. The presence of water inside LNPs has been suggested also by previous experimental calorimetric studies on LNP (made with a more complex mixture of lipids) [6]. It is interesting to note that water molecules inside the LNP are continuously exchanged with water molecules outside the LNP. This process is illustrated in Figure 5. As seen in that Figure, we observe that individual water molecules from inside or outside cross the core of the LNP. In that way, the water droplet inside the LNP is maintained in equilibrium with water outside the LNP.

**Figure 6:**
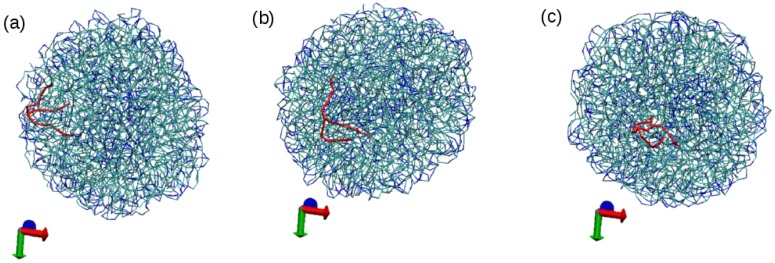
Snapshots of simulation S2 at different times to show the motion of tripalmitin lipid molecules in the NP. Tripalmitin molecules are displayed in bond representation, with non polar tails in cyan and polar heads in blue. A particular tripalmitin molecule (a “tracer” molecule) is shown in red in order to follow its motion during the simulation. (**a**) the tracer is at the surface of the NP in contact with water (631,000 simulation steps, *t* = 12.62 ns), (**b**) the tracer has moved towards the core of the NP (4,579,000 simulation steps, *t* = 91.58 ns), (**c**) the tracer molecule changes its orientation in the NP core (5,000,000, *t* = 100 ns) (water molecules are not shown in order to facilitate visualization).

At this point, we have to emphasize here that the lipid molecules inside the LNPs are also highly mobile. They can easily move from the surface of the NP to the core and can also easily change orientation, as illustrated in Figure 6 for simulation S2.

### 3.2. Effect of Surfactant

We have also performed simulations adding Tween 20 surfactant to the LNP. These simulations are motivated by previous experimental works [8], in which surfactant is added in order to stabilize the NPs. However, the actual effect of the surfactant on the NPs structure is not clear. It has been suggested that the adsorption of surfactant deforms the shape of the LNP. It is also not clear whether the surfactant adsorbs uniformly on the surface of the LNP or it tends to form aggregates adsorbed on the surface of the LNP. In order to clarify these questions, we have performed a series of simulations (S4 and S5 in Table 1) with different amounts of added Tween 20 surfactant.

**Figure 7:**
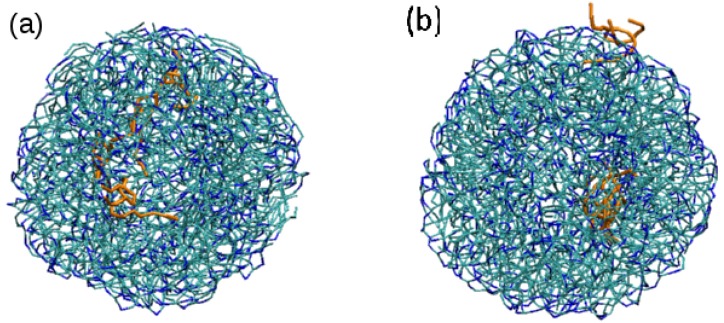
Snapshots from simulation S4 at different times to show the motion of Tween 20 molecules from the core of the LNP towards the surface: (**a**) snapshot obtained after 75,000 time steps (*t* = 1.5 ns); (**b**) snapshot obtained after 16,385,000 time steps (*t* = 327.7 ns). Both surfactant and lipid molecules are shown in bond representation, with Tween 20 molecules emphasized with an orange colour (blue and cyan colours correspond to polar and apolar beads of tripalmitin molecule, as in previous figures). Water molecules are not shown for clarity.

First, we performed a new simulation (S4) consisting of a repetition of simulation S2 (LNP of intermediate size) but with three Tween 20 molecules. The initial condition was the same as in simulation S2 but with the three surfactant molecules also included. Initially (after 1.5 ns), the NP self-assemble in a spherical NP in which the three Tween 20 molecules were found inside the NP, not at the surface (see Figure 7). The surfactant molecules were observed to freely diffuse inside the NP and, after a simulation time t≈328 ns, the surfactant molecules reached the NP surface and stayed at the surface during the remaining simulation time (see Figure 7).

**Figure 8:**
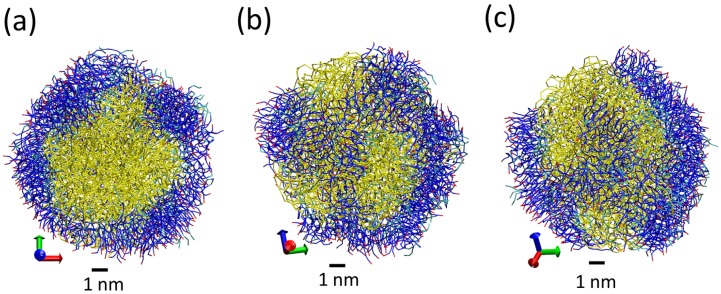
Snapshot from simulation S5 illustrating the inhomogeneous distribution of surfactant onto LNP. Tripalmitin lipid molecules are shown in yellow to facilitate discrimination of the lipid core of the LNP. Colours in Tween 20 molecules correspond to the different Martini beads: polar P${}_{3}$ beads are shown in red, hydrophilic N${}_{a}$ beads in blue and hydrophobic C${}_{1}$ in cyan. Each panel (**a**–**c**) corresponds to a different orientation of the same configuration. In all panels, surfactant and lipid molecules are shown in bond representation and water molecules are not shown for clarity.

Starting from the previously assembled NP, we have performed additional simulations adding more Tween 20 surfactant molecules. After several partial simulations adding more Tween 20 surfactant, we performed a final simulation with a large amount of Tween 20 (simulation with code S5 in Table 1). This simulation contains 221 surfactant molecules, which is a slightly larger amount than the number of tripalmitin lipid molecules.Snapshots from the simulations are seen in Figure 8. All surfactant molecules are found on the surface of the LNP (not inside the LNP), and are observed to be highly mobile. As seen in Figure 8, the surfactant molecules have a tendency to orient their polar groups in contact with water or in contact with hydrophilic groups of the lipid. We have computed in this case the radius of gyration for the total LNP (accounting for both lipids and surfactants) and for the lipid core (taking into account only lipid molecules). The obtained result was $R_{g}$ = 3.9 nm for the lipid core and $R_{g}=4.9$ nm for the total LNP. The $R_{g}$ obtained for the lipid core is slightly larger than the one obtained without surfactant addition for the same number of lipid molecules (see simulation S2 in Table 2). The difference between the total value of the $R_{g}$ and that of the lipid core indicates that the addition of surfactant increases substantially the size of the LNP. However, the surfactant does not form a well defined monolayer over the LNP. As seen in Figure 8a, the surface of the LNP has large regions without any surfactant coverage and regions with a thick layer of surfactant (in some places, it has a thickness of 2–3 nm, as seen in Figure 8a). In addition, the surfactant layer forms structures similar to strips on the LNP surface (see Figure 8b,c). In spite of the large amount of surfactant employed here, the LNP is not fully covered by surfactant molecules due to the formation of these thick layers of surfactants. Our results indicate that, at the molar fraction considered in our simulations, the surfactant layer cannot be considered a good protective layer for the LNP since its distribution is highly inhomogeneous as shown in Figure 8. We do not know whether an increase of the concentration within realistic molar ratios will be able to produce a layer of surfactant covering all the LNP. Further simulations are needed to clarify this point. In any case, our results indicate that the surfactant layer is not a single molecule layer and that the surfactant forms more complex structures than the simple monolayers suggested previously (compare, for example, our results in Figure 8 with the cartoon in Figure 10 of Ref. [8]).

## 4. Conclusions

In this work, we have performed molecular dynamics simulations of lipid nanoparticles (LNPs) made of tripalmitin lipid with and without added Tween 20 surfactant using the Martini coarse grain model. Our simulations were done at 310 K, which is the temperature of interest for biomedical applications. Our simulations show that lipids inside LNPs are highly mobile and poorly organized, so, in general, the LNP resembles more a liquid droplet than a self-assembled colloid (such as micelles or vesicles which show a high degree of organization). We observe a tendency of hydrophilic groups to be at the LNP external surface, but we also found hydrophilic groups from lipids inside the core of LNPs (which sometimes form small clusters of hydrophilic groups). We also found the formation of a water droplet inside the largest simulated LNP (a water droplet of about ≈2–3 nm inside an LNP of about ≈10 nm size). Interestingly, we observe that water is able to be exchanged between the interior and exterior of the LNP by diffusion of individual water molecules across the lipid core of the nanoparticle. Our results suggest that, in applications of LNP at physiological temperature, LNPs are able to encapsulate not only hydrophobic molecules but also hydrophilic molecules in their interior and to exchange their contents with their surrounding environment. Further simulation studies of encapsulation of molecules by LNP will be highly desirable in order to analyse this possibility in detail. Given that LNPs at 310 K are more a liquid droplet than a self-assembled nanoparticle, one may question whether the classical strategy of stabilizing particles with an adsorbed surfactant layer would be suitable for LNPs. Our simulations with added Tween 20 surfactant indicate that the surfactant tends to assemble at the surface of the LNP instead of covering the LNP with a homogeneous layer. The adsorbed surfactant layer is therefore more complex than a simple surfactant monolayer. Further simulations are required in order to determine whether it is possible or not to completely cover the LNP by using amounts of surfactant much larger than those considered here. The results obtained here demonstrate the potential of the Martini coarse-grain model in the study of lipid nanoparticles. Now, the most pressing issue to be analysed theoretically in this field concerns the study of the solid phase of LNPs obtained at low temperatures. In practice, LNPs are often prepared and stored at low temperatures to ensure a solid phase for the lipids (the so-called solid lipid nanoparticles, SLNP), which prevents delivery of active components trapped inside the SLNPs and also prevents coalescence of the nanoparticles in larger particles. The LNPs are expected to be liquid at physiological temperature (as seen here in our simulations), so they are able to release their active components. Now, the challenge will be to predict by simulations the particular solid lipid phases expected in the SLNPs. This is an important question in the performance of SLNPs (see, for example, Ref. [7]), which we hope to address in the near future.

## Figures and Tables

**Table 1:** Composition of simulations considered in this work (number of tripalmitin lipid molecules, number of Tween 20 surfactant molecules, and number of P${}_{4}$ water and AF beads). We also indicate the equilibrium value for the side *L* of the cubic simulation box (L×L×L) and the total simulation time for each case. In all cases, we have a temperature of 310 K and pressure of 1 atm.

	System	N Lipid	N Surfactant	N Water	AF Beads	*L*	Sim Time
S1	pure lipid NP	64	-	4176	462	9.2 nm	1100 ns
S2	pure lipid NP	216	-	28,003	3143	16.9 nm	100 ns
S3	pure lipid NP	392	-	60,210	6627	21.5 nm	450 ns
S4	surfacted NP	216	3	22,268	2420	15.6 nm	327.6 ns
S5	surfacted NP	216	221	31,826	3518	17.9 nm	201.8 ns

**Table 2:** Results from simulations of tripalmitin NP at 310 K and 1 atm. *N* is the number of tripalminitin molecules in the NP, $R_{g}$ is the radius of gyration (see Equation (1)), $3N/4\pi R_{g}^{3}$ is the number of lipid molecules per unit volume of the NP and Δ is the dimensionless asphericity parameter (see Equation (2)). All quantities are averages over equilibrium configurations and all statistical errors are smaller than the last significant figure.

Simulation	*N*	$R_{g}$	$3N/4\pi R_{g}^{3}$	Δ
S1	64	2.5 nm	0.98 nm${}^{-3}$	$1.2\times 10^{-3}$
S2	216	3.7 nm	1.02 nm${}^{-3}$	$8.2\times 10^{-4}$
S3	392	4.6 nm	0.96 nm${}^{-3}$	$7.8\times 10^{-4}$

## Abbreviations

The following abbreviations are used in this manuscript:

CG	Coarse grained model
CPK	Corey, Pauling and Koltun’s convention for the representation of atoms in molecules
CTAB	Cetrimonium bromide also known as cetyltrimethylammonium bromide or hexadecyltrimethylammonium bromide
LNP	Lipid NanoParticles
MD	Molecular Dynamics
SDS	Sodium dodecyl sulfate, also known as sodium lauryl sulfate
